# 5-Amino-7-(4-bromo­phen­yl)-3,7-di­hydro-2*H*-thieno[3,2-*b*]pyran-6-carbo­nitrile 1,1-dioxide

**DOI:** 10.1107/S1600536809055214

**Published:** 2010-01-09

**Authors:** Chen-Xia Yu, Xiao-Dong Feng, Bei Jiang, Cui-Hua Wang, Chang-Sheng Yao

**Affiliations:** a School of Chemistry and Chemical Engineering, Xuzhou Normal University, Xuzhou 221116, People’s Republic of China, and, Key Laboratory of Biotechnology for Medicinal Plants, Xuzhou Normal University, Xuzhou 221116, People’s Republic of China

## Abstract

In the title compound, C_14_H_11_BrN_2_O_3_S, the 2,3-dihydro­thio­phene ring is almost planar [maximum deviation = 0.006 (1) Å]. The pyran ring is in an envelope conformation [puckering parameters *Q* = 0.115 (2) Å, θ = 77.5 (10), ϕ = 172.9 (10)°]. The pyran and phenyl rings are approximately perpendicular, making a dihedral angle of −76.4 (2)°. The crystal packing is stabilized by inter­molecular N—H⋯O hydrogen bonds, with the sulfone O atoms acting as acceptors.

## Related literature

For the use of thienopyranyl compounds, such as thieno[3,2-*b*]pyran derivatives, as anti­viral agents, see: Friary *et al.* (1991 ▶) and as α-2C adrenoreceptor agonists, see: Chao *et al.* (2009 ▶). For puckering parameters, see: Cremer & Pople (1975 ▶).
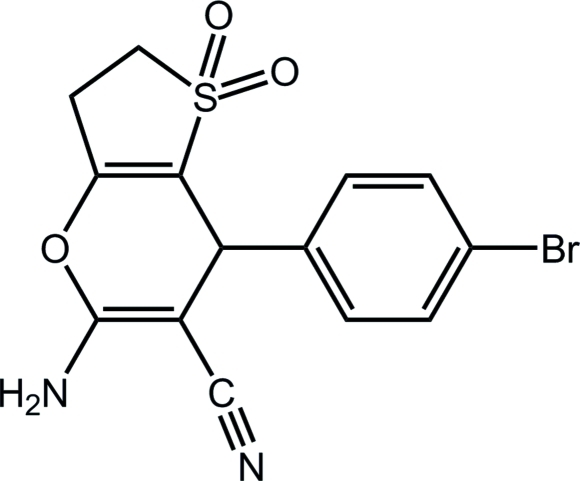


## Experimental

### 

#### Crystal data

C_14_H_11_BrN_2_O_3_S
*M* *_r_* = 367.22
Monoclinic, 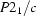

*a* = 8.3743 (18) Å
*b* = 14.003 (3) Å
*c* = 12.673 (3) Å
β = 103.059 (3)°
*V* = 1447.7 (5) Å^3^
*Z* = 4
Mo *K*α radiation
μ = 2.99 mm^−1^
*T* = 113 K
0.24 × 0.22 × 0.12 mm

#### Data collection

Rigaku Saturn CCD area-detector diffractometer
Absorption correction: multi-scan (*CrystalClear*; Rigaku/MSC, 2005 ▶) *T*_min_ = 0.534, *T*_max_ = 0.715
14473 measured reflections
3440 independent reflections
2636 reflections with *I* > 2σ(*I*)
*R*_int_ = 0.038

#### Refinement

*R*[*F* ^2^ > 2σ(*F* ^2^)] = 0.036
*wR*(*F* ^2^) = 0.088
*S* = 0.99
3440 reflections
198 parameters
H atoms treated by a mixture of independent and constrained refinement
Δρ_max_ = 0.43 e Å^−3^
Δρ_min_ = −0.95 e Å^−3^

### 

Data collection: *CrystalClear* (Rigaku/MSC, 2005 ▶); cell refinement: *CrystalClear*; data reduction: *CrystalClear*; program(s) used to solve structure: *SHELXS97* (Sheldrick, 2008 ▶); program(s) used to refine structure: *SHELXL97* (Sheldrick, 2008 ▶); molecular graphics: *SHELXTL* (Sheldrick, 2008 ▶); software used to prepare material for publication: *SHELXTL*.

## Supplementary Material

Crystal structure: contains datablocks I, global. DOI: 10.1107/S1600536809055214/fj2262sup1.cif

Structure factors: contains datablocks I. DOI: 10.1107/S1600536809055214/fj2262Isup2.hkl

Additional supplementary materials:  crystallographic information; 3D view; checkCIF report

## Figures and Tables

**Table 1:** Hydrogen-bond geometry (Å, °)

*D*—H⋯*A*	*D*—H	H⋯*A*	*D*⋯*A*	*D*—H⋯*A*
N2—H1⋯O2^i^	0.88 (3)	2.26 (3)	2.989 (3)	140 (2)
N2—H2⋯O3^ii^	0.91 (3)	2.02 (3)	2.919 (3)	169 (2)

Symmetry codes: (i) 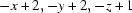; (ii) 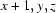.

**Fig. 1.**
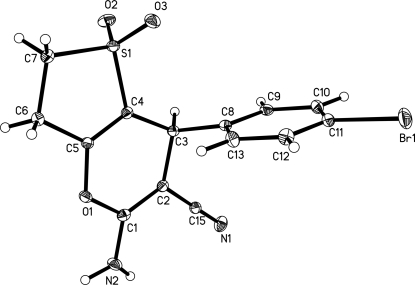
The structure of (I), showing 30% probability displacement ellipsoids and the atom-numbering scheme.

**Fig. 2.**
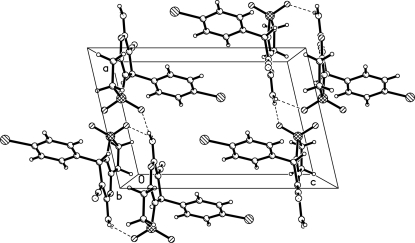
The packing diagram of (I). Intermolecular hydrogen bonds are shown as dashed lines.

## supplementary crystallographic information

### Comment

Thienopyranyl compounds, such as thieno [3,2-*b*]pyran derivatives, can be used as antiviral agents (Friary *et al.*, 1991) and α-2 C adrenoreceptor agonists (Chao *et al.*, 2009). This led us to pay much attention to the synthesis and bioactivity of these compounds. During the synthesis of thieno[3,2-*b*]pyran derivatives, the title compound, (I) was isolated and its structure was determined by X-ray diffraction. Here we shall report its crystal structure.

The molecular structure of (I) is shown in Fig. 1. In the molecular structure, the thiophene ring is in planar conformation, for the maximum deviation of C6 from the C4/C5/C6/C7/S1 plane is 0.006 (1) Å. For its weighted average *ABS*. torsion Angl. is 0.60°, less than 5.0°, Cremer & Pople puckering analysis was not performed(Cremer & Pople, 1975). The pyran ring adopts an envelope conformation with atome C3 deviating from the O1/C1/C2/C4/C5 plane 0.158 (3) Å. According to Cremer & Pople analysis, the puckering amplitude (Q) is 0.115 (2) Å. Its θ and φ are 77.5 (10) and 172.9 (10)°, respectively. The connection of the pyran ring and phenyl ring C8—C13 can be described by the C2—C3—C8—C13 torsion angle of -76.4 (2)°. The crystal packing is stablized intermolecular hydrogen bonds: N2—H1···O2, N2—H2···O3(Fig.2 & Table 1).

### Experimental

The title compound was synthesized by the reaction of dihydrothiophen-3(2*H*)-one-1,1-dioxide (1 mmol) and 2-(4-bromo benzylidene)malononitrile (1 mmol) catalyzed by triethylamine (0.02 g) in 10 ml ethanol under reluxing until completion (monitored by TLC). Cooling the reaction mixture slowly gave single crystals suitable for X-ray diffraction.

### Refinement

The H atoms bonded to the amide N atom was located in a difference map and were refined freely. Other H atoms were placed in calculated positions, with C—H = 0.95 or 0.99 Å, and included in the final cycles of refinement using a riding model, with *U*_iso_(H) = 1.2*U*_eq_(parent atom).

### Crystal data

**Table:**

C_14_H_11_BrN_2_O_3_S	*F*(000) = 736
*M**_r_* = 367.22	*D*_x_ = 1.685 Mg m^−^^3^
Monoclinic, *P*2_1_/*c*	Mo *K*α radiation, λ = 0.71070 Å
*a* = 8.3743 (18) Å	Cell parameters from 4061 reflections
*b* = 14.003 (3) Å	θ = 2.2–27.9°
*c* = 12.673 (3) Å	µ = 2.99 mm^−^^1^
β = 103.059 (3)°	*T* = 113 K
*V* = 1447.7 (5) Å^3^	Block, colorless
*Z* = 4	0.24 × 0.22 × 0.12 mm

### Data collection

**Table:**

Rigaku Saturn CCD area-detector diffractometer	3440 independent reflections
Radiation source: rotating anode	2636 reflections with *I* > 2σ(*I*)
confocal	*R*_int_ = 0.038
Detector resolution: 7.31 pixels mm^-1^	θ_max_ = 27.9°, θ_min_ = 2.2°
ω and φ scans	*h* = −10→10
Absorption correction: multi-scan (*CrystalClear*; Rigaku/MSC, 2005)	*k* = −18→18
*T*_min_ = 0.534, *T*_max_ = 0.715	*l* = −16→16
14473 measured reflections	

### Refinement

**Table:**

Refinement on *F*^2^	Primary atom site location: structure-invariant direct methods
Least-squares matrix: full	Secondary atom site location: difference Fourier map
*R*[*F*^2^ > 2σ(*F*^2^)] = 0.036	Hydrogen site location: inferred from neighbouring sites
*wR*(*F*^2^) = 0.088	H atoms treated by a mixture of independent and constrained refinement
*S* = 0.99	*w* = 1/[σ^2^(*F*_o_^2^) + (0.053*P*)^2^] where *P* = (*F*_o_^2^ + 2*F*_c_^2^)/3
3440 reflections	(Δ/σ)_max_ = 0.002
198 parameters	Δρ_max_ = 0.43 e Å^−^^3^
0 restraints	Δρ_min_ = −0.95 e Å^−^^3^

### Special details

**Table:**

Geometry. All e.s.d.'s (except the e.s.d. in the dihedral angle between two l.s. planes) are estimated using the full covariance matrix. The cell e.s.d.'s are taken into account individually in the estimation of e.s.d.'s in distances, angles and torsion angles; correlations between e.s.d.'s in cell parameters are only used when they are defined by crystal symmetry. An approximate (isotropic) treatment of cell e.s.d.'s is used for estimating e.s.d.'s involving l.s. planes.
Refinement. Refinement of *F*^2^ against ALL reflections. The weighted *R*-factor *wR* and goodness of fit *S* are based on *F*^2^, conventional *R*-factors *R* are based on *F*, with *F* set to zero for negative *F*^2^. The threshold expression of *F*^2^ > σ(*F*^2^) is used only for calculating *R*-factors(gt) *etc*. and is not relevant to the choice of reflections for refinement. *R*-factors based on *F*^2^ are statistically about twice as large as those based on *F*, and *R*- factors based on ALL data will be even larger.

### Fractional atomic coordinates and isotropic or equivalent isotropic displacement parameters (Å^2^)

**Table:**

	*x*	*y*	*z*	*U*_iso_*/*U*_eq_	
Br1	0.66967 (3)	1.11722 (2)	1.066878 (18)	0.03931 (11)	
S1	0.65228 (6)	0.83655 (4)	0.56887 (4)	0.01756 (13)	
O1	1.12195 (16)	0.86398 (10)	0.63555 (11)	0.0190 (3)	
O2	0.56187 (19)	0.87395 (11)	0.46675 (13)	0.0269 (4)	
O3	0.56402 (17)	0.83246 (11)	0.65464 (12)	0.0248 (4)	
N1	1.1137 (2)	1.20266 (14)	0.67001 (15)	0.0277 (4)	
N2	1.3147 (2)	0.97623 (16)	0.66289 (15)	0.0231 (4)	
C1	1.1535 (2)	0.95981 (16)	0.64815 (15)	0.0181 (4)	
C2	1.0334 (2)	1.02597 (15)	0.64622 (15)	0.0164 (4)	
C3	0.8526 (2)	1.00119 (14)	0.63725 (15)	0.0161 (4)	
H3	0.7842	1.0381	0.5758	0.019*	
C4	0.8376 (2)	0.89711 (14)	0.61035 (16)	0.0159 (4)	
C5	0.9603 (2)	0.83720 (15)	0.61122 (15)	0.0158 (4)	
C6	0.9284 (2)	0.73518 (15)	0.58117 (16)	0.0187 (4)	
H6A	0.9776	0.6932	0.6429	0.022*	
H6B	0.9761	0.7185	0.5189	0.022*	
C7	0.7412 (2)	0.72300 (15)	0.55110 (18)	0.0237 (5)	
H7A	0.7068	0.6746	0.5984	0.028*	
H7B	0.7050	0.7017	0.4750	0.028*	
C8	0.7996 (2)	1.02716 (15)	0.74159 (15)	0.0165 (4)	
C9	0.7130 (2)	1.11041 (14)	0.74657 (17)	0.0189 (4)	
H9	0.6812	1.1491	0.6838	0.023*	
C10	0.6716 (3)	1.13834 (17)	0.84266 (17)	0.0229 (5)	
H10	0.6122	1.1957	0.8460	0.027*	
C11	0.7190 (2)	1.08054 (17)	0.93298 (16)	0.0217 (5)	
C12	0.8041 (3)	0.99677 (16)	0.92947 (16)	0.0227 (5)	
H12	0.8346	0.9579	0.9922	0.027*	
C13	0.8450 (2)	0.96971 (15)	0.83348 (16)	0.0206 (5)	
H13	0.9037	0.9121	0.8304	0.025*	
C15	1.0788 (3)	1.12338 (16)	0.65907 (16)	0.0186 (4)	
H1	1.349 (3)	1.035 (2)	0.655 (2)	0.038 (7)*	
H2	1.382 (3)	0.925 (2)	0.659 (2)	0.038 (7)*	

### Atomic displacement parameters (Å^2^)

**Table:**

	*U*^11^	*U*^22^	*U*^33^	*U*^12^	*U*^13^	*U*^23^
Br1	0.03575 (17)	0.0621 (2)	0.02199 (14)	0.01328 (12)	0.01055 (11)	−0.00769 (11)
S1	0.0142 (3)	0.0163 (3)	0.0212 (3)	−0.0009 (2)	0.0022 (2)	−0.0001 (2)
O1	0.0143 (7)	0.0179 (8)	0.0251 (8)	0.0003 (6)	0.0051 (6)	−0.0008 (6)
O2	0.0224 (8)	0.0267 (9)	0.0271 (9)	−0.0035 (6)	−0.0042 (7)	0.0044 (7)
O3	0.0206 (8)	0.0226 (9)	0.0341 (9)	−0.0026 (7)	0.0121 (7)	−0.0002 (7)
N1	0.0360 (11)	0.0233 (11)	0.0261 (10)	−0.0079 (9)	0.0119 (8)	−0.0034 (8)
N2	0.0150 (9)	0.0235 (11)	0.0325 (11)	−0.0019 (9)	0.0089 (8)	0.0004 (9)
C1	0.0185 (10)	0.0226 (12)	0.0133 (9)	−0.0038 (9)	0.0037 (8)	0.0007 (8)
C2	0.0178 (10)	0.0174 (11)	0.0148 (9)	−0.0032 (8)	0.0054 (8)	−0.0011 (8)
C3	0.0144 (10)	0.0173 (11)	0.0163 (9)	−0.0010 (8)	0.0028 (8)	−0.0001 (8)
C4	0.0172 (10)	0.0164 (11)	0.0144 (9)	−0.0015 (8)	0.0042 (8)	−0.0004 (8)
C5	0.0134 (9)	0.0186 (11)	0.0154 (10)	−0.0001 (8)	0.0030 (8)	−0.0003 (8)
C6	0.0183 (10)	0.0188 (11)	0.0195 (10)	0.0012 (9)	0.0055 (8)	−0.0012 (8)
C7	0.0198 (11)	0.0182 (12)	0.0327 (12)	−0.0015 (9)	0.0052 (9)	−0.0055 (9)
C8	0.0139 (9)	0.0174 (11)	0.0182 (10)	−0.0025 (8)	0.0038 (8)	−0.0009 (8)
C9	0.0142 (10)	0.0202 (12)	0.0207 (10)	0.0014 (8)	0.0006 (8)	0.0011 (8)
C10	0.0186 (10)	0.0239 (12)	0.0254 (12)	0.0063 (9)	0.0036 (9)	−0.0042 (9)
C11	0.0176 (10)	0.0303 (13)	0.0183 (10)	−0.0013 (9)	0.0062 (8)	−0.0048 (9)
C12	0.0269 (11)	0.0210 (12)	0.0208 (10)	−0.0010 (9)	0.0069 (9)	0.0035 (9)
C13	0.0245 (11)	0.0159 (11)	0.0222 (11)	0.0051 (9)	0.0071 (9)	0.0040 (8)
C15	0.0180 (10)	0.0243 (13)	0.0146 (10)	−0.0017 (9)	0.0060 (8)	−0.0010 (8)

### Geometric parameters (Å, °)

**Table:**

Br1—C11	1.905 (2)	C4—C5	1.324 (3)
S1—O2	1.4421 (16)	C5—C6	1.487 (3)
S1—O3	1.4470 (15)	C6—C7	1.537 (3)
S1—C4	1.742 (2)	C6—H6A	0.9900
S1—C7	1.791 (2)	C6—H6B	0.9900
O1—C1	1.370 (3)	C7—H7A	0.9900
O1—C5	1.371 (2)	C7—H7B	0.9900
N1—C15	1.148 (3)	C8—C9	1.382 (3)
N2—C1	1.340 (3)	C8—C13	1.396 (3)
N2—H1	0.88 (3)	C9—C10	1.395 (3)
N2—H2	0.91 (3)	C9—H9	0.9500
C1—C2	1.364 (3)	C10—C11	1.385 (3)
C2—C15	1.416 (3)	C10—H10	0.9500
C2—C3	1.532 (3)	C11—C12	1.379 (3)
C3—C4	1.496 (3)	C12—C13	1.389 (3)
C3—C8	1.531 (3)	C12—H12	0.9500
C3—H3	1.0000	C13—H13	0.9500
			
O2—S1—O3	115.77 (10)	C7—C6—H6A	110.4
O2—S1—C4	110.20 (10)	C5—C6—H6B	110.4
O3—S1—C4	111.35 (9)	C7—C6—H6B	110.4
O2—S1—C7	111.00 (10)	H6A—C6—H6B	108.6
O3—S1—C7	110.89 (10)	C6—C7—S1	107.46 (14)
C4—S1—C7	95.89 (9)	C6—C7—H7A	110.2
C1—O1—C5	116.68 (16)	S1—C7—H7A	110.2
C1—N2—H1	119.2 (17)	C6—C7—H7B	110.2
C1—N2—H2	117.9 (17)	S1—C7—H7B	110.2
H1—N2—H2	120 (2)	H7A—C7—H7B	108.5
N2—C1—C2	127.0 (2)	C9—C8—C13	119.66 (18)
N2—C1—O1	110.25 (18)	C9—C8—C3	119.83 (18)
C2—C1—O1	122.75 (18)	C13—C8—C3	120.44 (18)
C1—C2—C15	118.32 (18)	C8—C9—C10	120.9 (2)
C1—C2—C3	124.07 (19)	C8—C9—H9	119.6
C15—C2—C3	117.53 (17)	C10—C9—H9	119.6
C4—C3—C8	113.76 (16)	C11—C10—C9	118.4 (2)
C4—C3—C2	105.54 (16)	C11—C10—H10	120.8
C8—C3—C2	110.88 (15)	C9—C10—H10	120.8
C4—C3—H3	108.8	C12—C11—C10	121.63 (19)
C8—C3—H3	108.8	C12—C11—Br1	118.42 (16)
C2—C3—H3	108.8	C10—C11—Br1	119.95 (17)
C5—C4—C3	126.21 (19)	C11—C12—C13	119.48 (19)
C5—C4—S1	109.30 (16)	C11—C12—H12	120.3
C3—C4—S1	124.47 (15)	C13—C12—H12	120.3
C4—C5—O1	123.58 (19)	C12—C13—C8	119.9 (2)
C4—C5—C6	120.83 (18)	C12—C13—H13	120.0
O1—C5—C6	115.56 (17)	C8—C13—H13	120.0
C5—C6—C7	106.51 (16)	N1—C15—C2	179.0 (2)
C5—C6—H6A	110.4		
			
C5—O1—C1—N2	−175.02 (16)	C1—O1—C5—C4	−7.0 (3)
C5—O1—C1—C2	5.5 (3)	C1—O1—C5—C6	170.86 (16)
N2—C1—C2—C15	1.2 (3)	C4—C5—C6—C7	1.2 (3)
O1—C1—C2—C15	−179.45 (17)	O1—C5—C6—C7	−176.74 (16)
N2—C1—C2—C3	−175.30 (19)	C5—C6—C7—S1	−0.9 (2)
O1—C1—C2—C3	4.1 (3)	O2—S1—C7—C6	114.83 (15)
C1—C2—C3—C4	−10.6 (2)	O3—S1—C7—C6	−115.01 (14)
C15—C2—C3—C4	172.93 (17)	C4—S1—C7—C6	0.54 (16)
C1—C2—C3—C8	113.0 (2)	C4—C3—C8—C9	−140.68 (18)
C15—C2—C3—C8	−63.5 (2)	C2—C3—C8—C9	100.6 (2)
C8—C3—C4—C5	−112.4 (2)	C4—C3—C8—C13	42.4 (2)
C2—C3—C4—C5	9.4 (3)	C2—C3—C8—C13	−76.4 (2)
C8—C3—C4—S1	69.5 (2)	C13—C8—C9—C10	0.6 (3)
C2—C3—C4—S1	−168.70 (14)	C3—C8—C9—C10	−176.30 (18)
O2—S1—C4—C5	−114.85 (15)	C8—C9—C10—C11	−0.1 (3)
O3—S1—C4—C5	115.27 (15)	C9—C10—C11—C12	−0.5 (3)
C7—S1—C4—C5	0.10 (17)	C9—C10—C11—Br1	178.53 (15)
O2—S1—C4—C3	63.5 (2)	C10—C11—C12—C13	0.6 (3)
O3—S1—C4—C3	−66.35 (19)	Br1—C11—C12—C13	−178.44 (16)
C7—S1—C4—C3	178.48 (17)	C11—C12—C13—C8	−0.1 (3)
C3—C4—C5—O1	−1.4 (3)	C9—C8—C13—C12	−0.5 (3)
S1—C4—C5—O1	176.96 (14)	C3—C8—C13—C12	176.40 (18)
C3—C4—C5—C6	−179.15 (18)	C1—C2—C15—N1	−156 (15)
S1—C4—C5—C6	−0.8 (2)	C3—C2—C15—N1	21 (15)

### Hydrogen-bond geometry (Å, °)

**Table:**

*D*—H···*A*	*D*—H	H···*A*	*D*···*A*	*D*—H···*A*
N2—H1···O2^i^	0.88 (3)	2.26 (3)	2.989 (3)	140 (2)
N2—H2···O3^ii^	0.91 (3)	2.02 (3)	2.919 (3)	169 (2)

Symmetry codes: (i) −*x*+2, −*y*+2, −*z*+1; (ii) *x*+1, *y*, *z*.
